# What can we learn from experiences in general practice during the COVID-19 pandemic? A qualitative study

**DOI:** 10.1186/s12913-023-09654-7

**Published:** 2023-06-27

**Authors:** Corinne Rijpkema, Nanne Bos, Daan Brandenbarg, Maarten Homburg, Gina Beugel, Wietske S. Barkema, Tim olde Hartman, Jean Muris, Lilian Peters, Marjolein Berger, Robert A. Verheij, Lotte Ramerman

**Affiliations:** 1 Netherlands Institute for Health Services Research, Nivel, Utrecht, The Netherlands; 2 Tilburg School of Social and Behavioral Sciences, Tilburg University, Tranzo, Tilburg, The Netherlands; 3 Department of Primary and Long-term Care, University of Groningen, University Medical Center Groningen, Groningen, The Netherlands; 4 Midwifery Science, Amsterdam University Medical Center, Vrije Universiteit Amsterdam, AVAG, Amsterdam Public Health, Amsterdam, The Netherlands; 5 Department of Primary and Community Care, Radboud University Nijmegen Medical Center, Radboud Institute for Medical Innovation, Nijmegen, The Netherlands; 6 Department of Family Medicine, CAPHRI Care and Public Health Research Institute, Maastricht University, Maastricht, The Netherlands

**Keywords:** COVID-19, General practice, Qualitative research, Care continuity, Appropriate care

## Abstract

**Background:**

Experiences with organizational changes in daytime general practices and out-of-hours (OOH) services during the COVID-19 pandemic may help to address the challenges in general practice care that were already a concern before the crisis. This study aimed to describe these experiences and the potential usefulness of the organizational changes for future general practice care and any future pandemics.

**Methods:**

Semi-structured interviews were performed among 11 directors of OOH services, and 19 (locum) general practitioners (GPs) or practice managers, who were purposively sampled. Video or telephone interviews were performed in two rounds: between November 2020 and January 2021 and between May 2021 and August 2021. The data were analyzed using thematic analysis methods.

**Results:**

Three themes emerged from the data: (1) *Changes in the triage procedures*; in GP practices and OOH services, stricter triage criteria were implemented, and GPs were more actively involved in the triage process. These measures helped to reduce the number of ‘low urgency’ face-to-face consultations. (2) *Changes in GP care*; there was a shift towards video and telephone consultations, allowing GPs to spend more time with patients during the remaining face-to-face consultations. For chronic patients, the shift towards telemonitoring appeared to encourage self-care, and postponing face-to-face consultations for regular checkups appeared to be unproblematic for stable patients. (3) *Coordination of GP care and information communication flow during the COVID-19 pandemic;* OOH directors perceived a lack of consistency in the information from various governmental and non-governmental parties on containment measures and guidelines related to COVID-19, making it difficult to act on them. The COVID-19 pandemic intensified collaboration between GPs, OOH services, and other healthcare professionals.

**Conclusions:**

The results of this study indicate that some of the organizational changes, such as stricter triage, remote consultations, and changes in managed care of chronic patients, may help in tackling the pre-existing challenges in GP care from before the COVID-19 pandemic. However, more extensive research and continuous monitoring are necessary to establish the effects on patients and their health outcomes. To navigate future pandemics, the intensified collaboration between health professionals should be maintained, while there is considerable room for improvement in the provision of unambiguous information.

**Supplementary Information:**

The online version contains supplementary material available at 10.1186/s12913-023-09654-7.

## Background

Before the COVID-19 pandemic, the core values of Dutch general practice (i.e. person-centered medical care, medical generalist care, continuity of care, and collaboration) had already been under great pressure [1]. The aging of the population has led to an increase in the consumption of care and the presentation of more complex health problems to the general practitioner (GP, also known as the family physician) [2]. This can lead to a decrease in the quality of care; effective, safe, and patient-centered care [3], because a higher workload is associated with a lower performance in general practice [4]. Furthermore, both chronic and preventive care have increasingly become the responsibility of GPs, whereas in the past this was provided by hospitals and outpatient clinics [1, 3]. Moreover, an increasing administrative burden and staff shortages have further increased the pressure on GP care [5]. This has all resulted in a perceived high workload among Dutch GPs, influencing the quality and accessibility of GP care [6].

The COVID-19 pandemic has had a large impact on healthcare systems, including primary care by general practitioners (GPs) during the working day and out of hours [7–10]. Due to the rapid spread of the COVID-19 virus, GPs were forced to change the organization of GP care [7, 8, 10, 11]. GPs postponed care that was not urgent or tried to provide care using safe (digital) alternatives to prevent the spread of COVID-19. Moreover, patients avoided primary care or treatment for fear of becoming infected [8, 12]. As a result, the organization of GP care changed dramatically, which impacted the core values of GP care [9].

During the COVID-19 pandemic, organizational changes were implemented to ensure accessibility and continuity of care by the GP. GPs’ experiences with the changes made during the pandemic may help in improving the accessibility, sustainability, and quality of primary health care provided by GPs after the COVID-19 pandemic and during any future pandemics [8, 13, 14]. Therefore, we aimed to describe the organizational changes implemented in GP care during the COVID-19 pandemic, evaluate the experiences of the healthcare professionals involved, and consider the viability and potential usefulness of the changes for future GP care and other pandemics.

General practitioner care in the Netherlands In the Netherlands, general practitioners are usually the first point of contact with a healthcare professional for any given health problem and they are the gatekeepers to specialized hospital care [3]. During office hours, GP care is provided by GP practices, with an average practice population of 2,095 individuals listed as patients in a practice. Managed care programs for the chronically ill are run by primary care groups, in which GPs play a central role. Practice personnel consist of one or more general practitioners and specialized support personnel who take care of relatively non-complex somatic and mental health problems [1, 15].

Out-of-hours services (OOH) with a catchment area of 450,000 people on average provide care for urgent health problems that cannot wait until the next working day [16]. Patients should first contact the OOH service by telephone. The telephone triage nurse assesses the urgency of the patient’s health problem and uses a triage protocol/system to determine the follow-up action: advice by phone, consultation at the OOH-GP location, or home visit by a GP.

Phases of the COVID-19 pandemic in the Netherlands The COVID-19 pandemic in the Netherlands can be described in different phases, based on containment measures and infection rates [17].

**Phase 0 (weeks 1–8) in 2020**

period before the COVID-19 pandemic.

**Phase 1 (weeks 9–24) in 2020**

first wave of COVID-19 infections – a lockdown was introduced with i.e. social distancing, working from home, and closing of schools/restaurants/sports facilities.

**Phase 2 (weeks 25–37) in 2020**

calmer period with fewer infections – limited containment measures i.e. social distancing.

**Phase 3 (weeks 38 − 16) in 2020/2021**

second wave of COVID-19 infections – a lockdown with additional containment measures i.e. use of face masks in public spaces, closing of non-essential stores, and an evening clock. Start of COVID-19 vaccination.

**Phase 4 (weeks 17–42) in 2021**

second calmer period with fewer infections – limited containment measures i.e. social distancing.

**Phase 5 (weeks 43–52) in 2021**

third wave of COVID-19 infections – a lockdown with additional containment measures i.e. COVID-19 access certificates for access to events/restaurants, introduction of self-testing.

## Methods

### Study setting and participants

In this descriptive qualitative study, a phenomenological approach was used, which enabled us to capture the experiences of the different healthcare professionals during the phenomenon studied: the organizational changes in GP care during the COVID-19 pandemic, both within office hours and out of hours [18]. For this study, we purposively selected GPs, GP practice managers, locum GPs and directors of OOH services [19]. In selecting these participants, attention was paid to the distribution of healthcare professionals, practice size, and region, to ensure inclusion of areas with low COVID-19 prevalence and areas with high COVID-19 prevalence. GPs and GP practice managers were recruited by contacting respondents to a previous questionnaire about changes in GP care during the COVID-19 pandemic, who indicated their willingness to participate in an interview [20]. Locum GPs and directors of OOH services were recruited through the personal and professional networks of the research team or via general contact information on websites of OOH services. The potential participants were contacted by telephone, after which an e-mail was sent to confirm the appointment and to provide additional information about the study and the interview procedure for those who wanted to participate. Three GPs refused to participate because of their increased workload due to the COVID-19 pandemic.

### Data collection

Two trained interviewers (CR, WSB) conducted two rounds of interviews: 16 interviews between November 2020 and January 2021, and 14 between May 2021 and August 2021. Two rounds of interviews were performed to take different phases of the pandemic into account and to adapt the topic list accordingly. There were no personal or professional relationships between participants and interviewers. The aim was to reach data saturation through the iterative process of interviewing, coding, and analyzing data until the richness and depth of the data were sufficient.

Semi-structured interviews were conducted using a topic list, which consisted of open-ended questions to gain in-depth perspectives. The topic list was constructed by members of the research team (CR, NB, DB, LR, LP, GB), and reviewed by the GPs in the research team (ToH, JM, MB) and an advisory board of 10 GPs. This provided insight into the important topics and gaps in knowledge in this area. The topic list consisted of five main topics: (1) organizational changes in GP care; (2) experiences with these organizational changes; (3) effects of the COVID-19 pandemic on the use of healthcare in specific patient groups; (4) quality and accessibility of care during the COVID-19 pandemic; and (5) reflection on the organizational changes’ potential usefulness for the future. At the end of the interview, the interviewees were free to add important topics to the discussion. After the first round of interviews, we reviewed the first results with GPs in the research team and advisory board and adjusted the topic list accordingly. An overview of the topic list is presented in Additional file 1.

The interviews were conducted using GoToMeeting, Zoom, or Microsoft Teams or by telephone, due to restrictions related to the COVID-19 pandemic. Only the participant and researcher were present during the interviews. The duration of the interviews ranged between 30 and 60 min. All interviews were audio-recorded and field notes were made by the interviewer. A summary of the interview was sent to all interviewees for approval to ensure the correct interpretation and thereby improve the reliability of our analysis (i.e. member check).

Participants were informed about the goal and nature of the study at the start of the interview and participated voluntarily without (financial) compensation. Participants were free to provide personal information (i.e. age, work experience) and could contact the researchers for access to, rectification of, or deletion of personal information. Data were only accessible by members of the research team and were processed anonymously.

### Data analysis

The audio recordings were transcribed verbatim. The transcripts were analyzed by two researchers (CR, GB) immediately after conducting the first interview. The six steps of Braun and Clarke were used for inductive thematic data analysis, where themes were extracted from the data [21, 22]. A thematic analysis was conducted in order to understand experiences, thoughts, or behaviors from the interviewees [21, 22]. The two researchers independently coded the first three interviews, after which the interviews were discussed. The remaining transcripts of the interviews were divided for coding (by CR or GB) and checked by the other researcher. During the first step of the coding process, the researchers read the interview transcripts in full to familiarize themselves with the data. Secondly, the researchers generated initial codes that describe features of potentially relevant data (open coding). If these codes differed between the researchers they were discussed until a consensus was reached. In the third step, the transcripts were searched for overarching themes, based on the topic list, which then provided the basis for the coding tree (axial coding). Fourthly, the themes were reviewed in relation to the data (selective coding), and the coding tree was discussed with GPs. In the fifth step, each theme name was described uniquely and specifically in a few sentences. Finally, the information related to the themes was compiled to give an overview of the findings on the perspectives of GP healthcare professionals regarding the viability and potential usefulness of the changes for future GP care and pandemics [21]. The steps in the data analysis process were iterative. The coding process was performed in Atlas.ti, versions 8.1 and 9 [23, 24]. For writing this report, we used the Consolidated criteria for Reporting Qualitative research (COREQ) checklist, see additional file 2 [25].

## Results

In total, 30 interviews were performed: 11 with directors of OOH services, 14 with GPs, 2 with locum GPs, and 3 with GP practice managers (Table 1).

**Table 1:** Characteristics of interviewees working in Dutch GP practices and OOH services

		GP practices (n=19)	OOH services (n = 11)
Sex			
Male		11	5
Female		8	6
Age (Range)		32–64 years	34–60 years
Region			
North		4	2
Middle		10	7
South		5	2
Type of practice			
Solo		5	-
Duo		5	-
Group		9	-
Responsible for … OOH-GP locations			
1		-	4
2		-	1
3		-	2
4		-	3
5		-	1

**Fig. 1:**
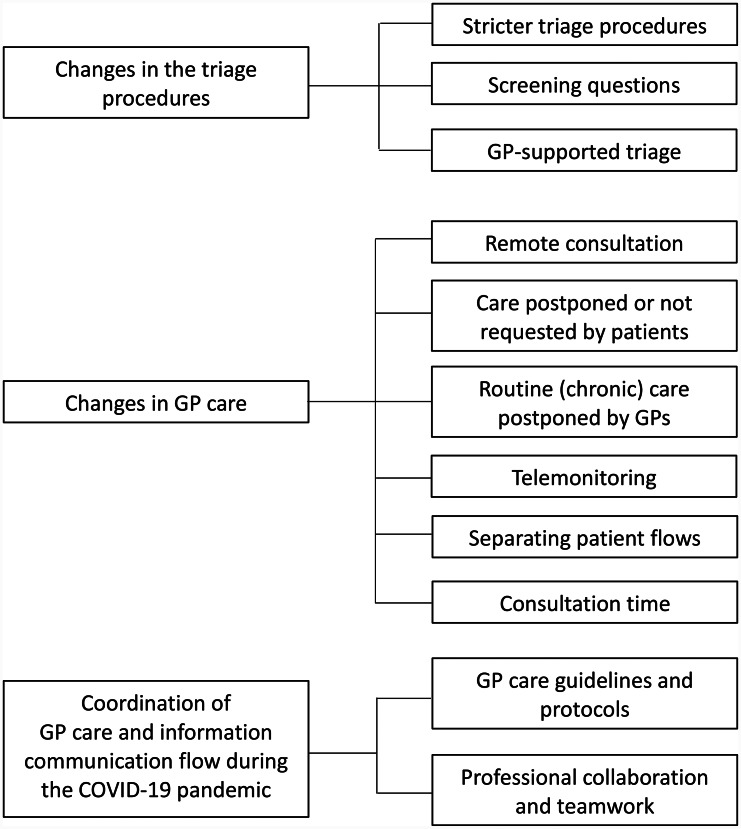
Themes and subthemes that emerged from the data

Three main themes were identified based on the organizational changes in GP care for patients due to the COVID-19 pandemic, viewed from a GP’s perspective (Fig. 1):

1. the changes in the triage procedures;
2. the changes in GP care;
3. the coordination of GP care and information communication flow during the COVID-19 pandemic.

### The changes in the triage procedures

Both GP practices and OOH services modified the procedure for triage prior to a consultation. GPs and OOH directors said that stricter triage criteria (the assignment of lower urgency levels) were adopted to make sure that face-to-face care was only provided when absolutely necessary. With stricter triage, people with low-urgency care needs (U4 - negligible chance of harm and U5 - no chance of harm) during out of hours were referred to their GP the next working day or were provided with advice by phone. Because of these stricter triage criteria, some GPs had conflicts with patients who would have preferred a face-to-face consultation. These GPs indicated that this resulted in reduced access to GP care for their patients. However, some of the OOH directors said that stricter triage improved the quality of care:

*“We applied stricter triage and I think, in the end, this led to a better quality of care. If patients visit the OOH service with no reason, then they should not come.”* – OOH service director (6).

Stricter triage and more advice by phone instead of in consultations at OOH services were considered by most OOH directors to be useful changes for the non-pandemic future to divert patients with low-urgency health problems who can wait for GP care during office hours.

GPs and OOH directors mentioned the introduction of additional triage questions aimed at preventing the spread of COVID-19 in GP practices and OOH services. These questions screened for influenza-like symptoms that could indicate COVID-19. Patients presenting with these symptoms were obliged to test for COVID-19 before visiting the GP practice or OOH service. Even though this was to protect patients from COVID-19, it resulted in reduced access to GP care for their patients, according to GPs and OOH directors. Interviewees mentioned that the duration of the triage process increased due to the more extended triage, which they thought had resulted in a higher workload for triage nurses and doctor’s assistants, as well as reduced accessibility for patients.

At some OOH services and GP practices, GPs became more involved in the triage to support the triage nurses and assistants if they were uncertain about something during triage and telephone consultations. OOH directors emphasized that this resulted in more care delivered remotely and a reduced workload for triage nurses and GPs. However, some interviewees also noticed downsides:

*“As a triage nurse you can handle things independently, but once there is a GP sitting next to you, you are much more inclined to ask things. So things you normally handle on your own, you now ask the GP and that takes time, while it is not always medically necessary.”* – OOH service director (and experienced triage nurse) (7).

At some OOH services, directors mentioned that it may be beneficial to involve GPs in triage more even after the COVID-19 pandemic, allowing more consultations to take place by telephone or video call and reducing the workload for GPs.

### The changes in GP care

Both in GP practices and OOH services, the interviewees reported an increase in remote care (e.g. e-consultation, or video consultation) during the COVID-19 pandemic. The interviewed GPs were satisfied with the use of remote consultations, explaining that many health problems can be diagnosed and discussed remotely, leaving more time for more complex health problems that require face-to-face consultations. In addition, they found that video consultations made it easier to reassure patients compared with telephone consultations, avoiding the need for a visit. GPs said that for the best quality of care, remote consultation and face-to-face consultations should be combined and the choice of remote consultations should be left to patients exclusively. Almost all interviewees indicated that they want to use remote consultations more frequently from now on:

*“In the future, we will have less staff, while the number of chronic patients is increasing. To cope with that change, you have to monitor your patients remotely […] that offers a lot of added value.”* – OOH service director (5).

However, some of the GPs did experience barriers in using remote consultations. They mentioned that the quality and accessibility of care decreased, specifically in some patient groups such as older adults, people in a lower socioeconomic status, and people with low health literacy. Additionally, GPs experienced a lack of non-verbal communication, and more difficulty in assessing symptoms and diagnoses. During the pandemic, remote consultations were essential for the continuation of GP care. However, in a non-pandemic situation, these GPs prefer face-to-face consultations as they can then carry out a physical examination of the patient.

During the COVID-19 pandemic, GPs experienced an overall decrease in the number of consultations. The interviewees noted that patients with minor complaints tended to avoid care for fear of becoming infected with COVID-19 or because they believed their GP was too busy. Consequently, several GPs feared that these patients would need more care later on. The effects were already visible for some GPs, as they noticed an increase in the number of double consultation times during a later phase of the pandemic. In addition, GPs also noticed that some patients did not visit the GP who would normally do so, for example people with mental health problems. As a result, some GPs feared the consequences of missed care for their patients. Besides the negative consequences of missed care, some of the GPs in this study mentioned that the COVID-19 pandemic also made patients more aware that some health problems disappear spontaneously.

*“The efficiency of GP care has improved. I also think it is important that patients do not visit the OOH service unnecessarily. That is better for themselves, but also for the care providers” –* OOH service director (1).

In addition to *patients* postponing or avoiding care, *GPs* also postponed care or provided care for their patients remotely. For example, most GPs stated that chronic care for patients with Chronic Obstructive Pulmonary Disease (COPD), diabetes, cardiovascular disease, and mental health problems was postponed. Some GPs reported that disrupted chronic care led to a deterioration in the health status of some patients. However, others felt that these consultations could be postponed for a short period without causing problems, especially when patients are stable. Half of the GPs introduced telemonitoring to continue some of the chronic care, whereby patients checked their blood pressure, glucose, or oxygen saturation themselves and reported this to their GP.

*“We gave people with chronic conditions the opportunity to do measurements at home and pass this on to the GP by telephone. It is possible to do regular check-ups remotely; however, these check-ups also have a clear added value, but technically you can advise people to measure their blood pressure themselves and pass this on by telephone.” –* General practitioner (2).

GPs reported that patients were satisfied with the telemonitoring because they became more independent in managing their disease. GPs who used telemonitoring all indicated that they would like to continue the use for patients with chronic conditions. However, to enable effective use, GPs suggested setting cut-off values for telemonitoring outcomes that would trigger a visit to the GP, in addition to less frequent regular checks.

*“I think that in the long-term we will also have to move towards telemonitoring with the entire preventive chronic care. That you will also have fewer face-to-face contact moments for chronic care and more telemonitoring of the patients. And that certain cut-off values require a visit to the GP.” –* General practitioner (4).

The interviewed GPs noticed the effects of postponed care in other areas of healthcare. They said that diagnostic tests, such as the breast cancer screening program and diagnostic pulmonary function tests, were suspended temporarily, resulting in late diagnosis and postponed treatment.

*“Normally we have breast cancer screening in July, and now we started last week (December) [.] you immediately have a woman who already has a BI-RADS IV. So on the scale from I to V, it is very high. Normally, we would have noticed that earlier.”* – General practitioner (3).

In order to provide safe care for all patients during the COVID-19 pandemic, patient flows were separated into COVID and non-COVID. In some regions, OOH directors and GPs emphasized that care for patients with COVID-19-like symptoms was organized centrally at an OOH service location or a single GP practice, because there was fear of contamination among GPs, shortages of personal protective equipment (PPE), staff shortages, and an increasing number of COVID patients.

*“Because we did not really know what was coming our way and we did not immediately have a lot of PPE available, we had to choose between COVID patients and non-COVID patients. We then chose to see the non-COVID patients in their practice from the very beginning and the COVID patients could go to an OOH service during the daytime, where a locum GP was available.”* – OOH service director (2).

Once GPs became more accustomed to the pandemic situation, care for patients with COVID-19-like symptoms became more decentralized, at the patient’s own GP practice. In some regions, care for COVID patients was organized in individual practices throughout the study period, as not all regions had a suitable location for centralizing COVID care. In these regions, GPs said that patients with COVID-19-like symptoms were separated from other patients in individual practices or were seen in a specific consultation room and during specific times. Some GPs mentioned that separating patient flows affected the accessibility and quality of regular GP care, as it was not always possible for patients to visit their GP.

Most GPs said that the consultation time in GP practices had been extended from 10 to 15–20 min, to prevent crowding in the waiting room. The interviewed GPs were happy with the extended consultation times, as it enabled them to pay more attention to their patients, which increased job satisfaction and improved the quality of GP care.

*“We have become accustomed to those fifteen minutes, it provides calmness. You can work effectively and consultations are not overloaded.”* – General practitioner (1).

Most GPs said they would continue allocating longer consultation times for their patients in the future. However, some GPs felt that this reduced the availability of care, as fewer patients could be seen by any given GP. As a consequence, waiting times for a consultation increased, which could result in unmet healthcare demands in the long term. A solution proposed by the GPs is 5-minute telephone consultations for simple health problems, combined with 15-minute face-to-face consultations for people with more complex health problems.

### Coordination of GP care and information communication flow during the COVID-19 pandemic

OOH directors said that they were members of the regional ‘crisis team’, in which new COVID-19 information and recommendations on a national level (i.e. from governmental parties or general practitioners’ associations) were translated into practical measures for the GP practices and OOH services. According to the OOH directors, they informed GPs in daily communications about new guidelines and protocols related to COVID-19: when does a patient have COVID-19, what does separating patient flows mean, and how can you ensure a safe working environment for employees? For the interviewed OOH directors, this created an enormous challenge in deciding what should be communicated, as there was a lot of conflicting information available from different parties (e.g. the Ministry of Health, the National Institute for Public Health, and the GP Association LHV). They felt that this made it difficult for them to properly disseminate information.

*“Every day there were new insights, new guidelines. So that was a very hectic organizational period during the first two months. That means that, as a crisis team, you have to manage something that nobody understands. Which we learned by doing.”* – OOH service director (1).

As a result, OOH directors noted that some GPs developed practice-specific guidelines rather than adopting the crisis team’s guidelines, resulting in further fragmentation. Most GPs and directors of OOH services specified that in the future there should be more clarity and less ambiguity in the guidelines provided by the different parties, to avoid discussion and convey a consistent message.

Most GPs and OOH directors said that the COVID-19 pandemic positively impacted the regional cooperation between GPs. According to the interviewees, continuing these improved lines of cooperation after the pandemic has added value for the quality of GP care. They emphasized that the quality of care was improved by enhanced communication between GPs, the crisis team, other primary care providers, and the hospital, resulting in the quick and easy exchange of information.

*“The COVID-19 pandemic has led to specialists contacting us (GPs) more and vice versa. There was more mutual understanding and that has led to more cooperation.”* – General practitioner (10).

In addition to this, GPs and doctors in hospitals had more regular contact about patient flows from primary to secondary care. Moreover, GPs jointly arranged the separation of patient flows. GPs and OOH directors would appreciate the continuation of the regional collaboration between GPs, hospitals, and other primary healthcare providers established during the pandemic.

## Discussion

This study explored the organizational changes in GP care during the COVID-19 pandemic, and evaluated the experiences of Dutch healthcare professionals in GP care and the potential usefulness for future GP care and any future pandemics. Three main themes emerged regarding the experiences; (1) the changes in the triage procedures; (2) the changes in GP care; and (3) the coordination of GP care and information communication flow during the COVID-19 pandemic.

To prevent the spread of the COVID-19 virus, face-to-face consultations were avoided when possible. Stricter and more comprehensive triage played an important role in this, as did the closer involvement of GPs in triage in OOH services. Triage is important in controlling patient flows and in providing appropriate care for patients [26]. Triage may become increasingly important to cope with the expected increase in demand for GP healthcare services due to the aging population, at a time when staff shortages are likely to persist. To enable stricter triage, triage nurses should receive more training [27] and triage protocols should be adapted to ensure that they are not ‘too safe’ so that patients end up unnecessarily using the OOH services [28]. However, one of the dangers of stricter triage is wrongly precluding patients from care, which may have far-reaching consequences for the health outcomes of these patients [29]. In addition to stricter triage, increased involvement of GPs in triage may help reduce the workload for triage nurses, improve patient satisfaction with advice provided during triage [30], and reduce the use of OOH services, which in turn can help reduce the workload for GPs themselves [31]. However, Campbell et al. suggested there could be an increased number of consultations as a result of GP-supported triage compared to consultations without a clear (protocolized) triage [32], and Graversen et al. suggested that GPs were less effective in triage than nurses [33]. When implementing stricter triage and GP-supported triage, these hurdles should be addressed.

GPs and OOH directors had mixed opinions about the accelerated implementation of remote consultations. A positive experience was that remote consultations for minor health problems freed up time for extensive face-to-face consultations for patients with more complex health problems. This effect was found in another study, in which remote consultations increased the continuity and efficiency of GP care, and also lowered the threshold for patients to consult the GP [34]. However, some GPs in the current study also experienced barriers that impacted the quality and accessibility of GP care and that are in line with previous studies. Examples were the lack of non-verbal communication (e.g. How does the patient walk into the practice? What is their breathing like?), the difficulty of diagnosing patients (and missing diagnoses) [35], and the fact that this solution is not suitable for certain patient groups [9, 36]. Furthermore, a study by Keuper et al. (2021) suggested that Dutch GPs do not plan to continue the intensive use of remote consultations after the COVID-19 pandemic because of the required workflow changes, the time that needs to be invested in training personnel and patients, and the substantial costs of implementing a remote consultation system [20, 37]. Despite the varying experiences, remote consultations are still a viable option to navigate the expected increase in workload due to the aging population, task shifting, and staff shortages in GP care [20, 38, 39]. However, the health outcomes of patients receiving remote consultations should be monitored continuously and GPs should keep in mind the barriers in patient care. As suggested by the participants in this study, the patient should always have a deciding say in whether a consultation is remote or face-to-face.

Due to the COVID-19 pandemic, care for patients with chronic diseases was postponed. GPs in the current study expressed concern about the deterioration of the chronic condition in some of their patients, a fear that has also been recognized in Belgium [9, 40]. The consequences of postponed chronic care due to the COVID-19 pandemic remain mostly unknown to date; therefore, future research should address the long-term consequences. This is especially important as previous pandemics showed that postponing regular health checks and secondary prevention, such as those provided by managed care programs, can lead to the aggravation of chronic diseases [41]. However, according to the current study, regular check-ups may not be required for all patients. GPs suggested that care for more stable patients could be postponed for a short period, relying more on self-management of their chronic disease, including via telemonitoring. Telemonitoring may increase access to healthcare and may help tackle the challenge of meeting the demand for healthcare [42]. Therefore, there is a need for research focused on identifying groups of patients with chronic conditions where it is safe to postpone care and groups where care should be continued, enabling a better allocation of more extensive managed care and telemonitoring solutions.

There were also organizational changes that may be useful for future pandemics, including the intensified collaboration between GPs and other healthcare professionals, which was also found during previous pandemics or epidemics [14]. The lack of unambiguous information is not a new phenomenon, and has been seen previously in pandemics [14]. Previous studies have also shown that during pandemics there is frequently (often contradictory) information coming from many different sources, and there are no clear routes for feedback on guidelines [43, 44]. In future pandemics, it is recommended to reduce duplication in information from different governmental parties, increase clarity in communication and provide clear and consistent guidance [45].

This study aimed to investigate GPs’ experiences with the provision of healthcare to their patient population. From an organizational perspective, the role GPs described was mainly focused on the continuity and accessibility of care, i.e. maintaining the core values of primary care as much as possible and at the same time providing care for COVID-19 patients, without endangering other patients. Lauriola et al. suggest, however, that GPs and other health professionals in primary care could play a more extensive part in future pandemics [46]. Because GPs are close to society, they can play a crucial role in monitoring the impact of the pandemic on society and implementing local solutions for community health [46]. Due to their focus on organizational changes during the pandemic, GPs may not have focused on the role of monitoring and implementing solutions to control the COVID-19 pandemic, such as diagnosis (screening), contact tracing, and the physical and psychological monitoring and management of patients. However, any more extensive role should take into account their already increasing workload and range of different tasks.

### Strengths and limitations

This study had some limitations that should be considered when interpreting the results. The findings of our study may not be fully generalizable to other countries with different healthcare systems and should, therefore, be interpreted from the perspective of the healthcare system in question. In addition, although we used purposive sampling to select a wide variety of interviewees and to reach data saturation, there is a possibility that some insights and experiences are missing. Due to the phenomenological design, the themes that emerged from the data are almost identical to the themes in the topic list. Lastly, the participants were recruited by contacting respondents from a previous questionnaire and through the professional and personal social networks of the research team, which may have contributed to selection bias for some of the participants.

This study also had some strengths that are worth mentioning. We included a variety of healthcare professionals who are representative of GP care, which ensured different perspectives on the organizational changes in GP care throughout the Netherlands, resulting in richness of the data. Another strength is that we interviewed healthcare professionals in two separate rounds during the pandemic, which made it possible to obtain perspectives on the different phases of the COVID-19 pandemic.

## Conclusion

During the COVID-19 pandemic, stricter triage, GP involvement in the triage, and remote consultations were either newly implemented or extended. These changes may be useful for the future in enabling GP care to cope with staff shortages, task shifting, and the increasing demand for healthcare due to the aging population. The changes can also be useful for strengthening the role of primary care in relation to secondary care to keep the Dutch healthcare system accessible, sustainable, and of sufficient quality. However, the effects on patient care and the health outcomes of these patients should be considered and the effects should be continuously monitored. Furthermore, it is important to continue monitoring the impact of postponed chronic care to consider whether routine managed care is useful for these patients. Future pandemics may benefit from intensive collaboration. Clarity in providing unambiguous information is desirable to maintain regular GP care and care for infected people during pandemics. Due to their role in society, GPs can be crucial in monitoring the impact of pandemics on society and implementing local solutions for community health; however, this should be considered in light of their already increasing range of tasks and workload.

## Electronic supplementary material

Below is the link to the electronic supplementary material.

Supplementary Material 1: Interview topic list GP care

## Data Availability

The raw data underlying this article will not be shared due to the anonymity of the participants. Reasonable requests for de-identified raw data will be considered by the corresponding author.

## List of Abbreviations

COVID-19: Coronavirus Disease 2019
COPD: Chronic Obstructive Pulmonary Disease
GP: General Practitioner
LHV: The National General Practitioner Association
OOH services: Out-of-hours services
PPE: Personal Protective Equipment
