# Supplementary material for: What can we learn from experiences in general practice during the COVID-19 pandemic? A qualitative study
Source: BMC Health Serv Res. 2023 Jun 27;23:696. doi: 10.1186/s12913-023-09654-7 (PMC10294327; doi:10.1186/s12913-023-09654-7)
Supplement: Supplementary file 1 — Supplementary Material 1: Interview topic list GP care [file 12913_2023_9654_MOESM1_ESM.docx]

**Appendix 1**

**Interview topic list GP care**

1. What organizational changes, as a result of the COVID-19 pandemic, took place within your general practice?
2. How did you experience these organizational changes, as a result of the COVID-19 pandemic, in your general practice?
3. What were the effects of these organizational changes on the use of care by different patient (groups)?
4. What were/are your experiences/perceptions regarding the quality of care for different patient (groups)?

- 4a. Can you explain the accessibility of care during the COVID-19 pandemic?
- 4b. What are your experiences with delayed care of different patient (groups)?
- 4c. In your opinion: can you explain how patients experienced the quality of care?

1. What organizational changes do you think have been effective during the first (and second) wave and will be maintained in future GP care?
2. Which organizational changes still need attention for future use in GP care?

*Note: the same questions were asked to directors of OOH services to obtain perspectives of organizational changes in OOH services.
